# Using knowledge graphs to infer gene expression in plants

**DOI:** 10.3389/frai.2023.1201002

**Published:** 2023-06-13

**Authors:** Anne E. Thessen, Laurel Cooper, Tyson L. Swetnam, Harshad Hegde, Justin Reese, Justin Elser, Pankaj Jaiswal

**Affiliations:** ^1^Department of Biomedical Informatics, University of Colorado Anschutz Medical Campus, Aurora, CO, United States; ^2^Department of Botany and Plant Pathology, Oregon State University, Corvallis, OR, United States; ^3^BIO5 Institute, University of Arizona, Tucson, AZ, United States; ^4^Environmental Genomics and Systems Biology Division, Berkeley Lab (DOE), Berkeley, CA, United States

**Keywords:** knowledge graph (KG), plant genome, gene expression, ontology, phenotype

## Abstract

**Introduction:**

Climate change is already affecting ecosystems around the world and forcing us to adapt to meet societal needs. The speed with which climate change is progressing necessitates a massive scaling up of the number of species with understood genotype-environment-phenotype (G×E×P) dynamics in order to increase ecosystem and agriculture resilience. An important part of predicting phenotype is understanding the complex gene regulatory networks present in organisms. Previous work has demonstrated that knowledge about one species can be applied to another using ontologically-supported knowledge bases that exploit homologous structures and homologous genes. These types of structures that can apply knowledge about one species to another have the potential to enable the massive scaling up that is needed through *in silico* experimentation.

**Methods:**

We developed one such structure, a knowledge graph (KG) using information from Planteome and the EMBL-EBI Expression Atlas that connects gene expression, molecular interactions, functions, and pathways to homology-based gene annotations. Our preliminary analysis uses data from gene expression studies in *Arabidopsis thaliana* and *Populus trichocarpa* plants exposed to drought conditions.

**Results:**

A graph query identified 16 pairs of homologous genes in these two taxa, some of which show opposite patterns of gene expression in response to drought. As expected, analysis of the upstream cis-regulatory region of these genes revealed that homologs with similar expression behavior had conserved cis-regulatory regions and potential interaction with similar trans-elements, unlike homologs that changed their expression in opposite ways.

**Discussion:**

This suggests that even though the homologous pairs share common ancestry and functional roles, predicting expression and phenotype through homology inference needs careful consideration of integrating cis and trans-regulatory components in the curated and inferred knowledge graph.

## Introduction

Climate change is already affecting ecosystems around the world and forcing us to explore ways to adapt to meet societal needs. This is particularly true in crop science where researchers are working to identify and predict genes and their resulting phenotypes under different environmental conditions in order to secure food production under a new climate regime (Thudi et al., 2021; Tian et al., 2021). Understanding gene/phenotype/environment relationships requires a large data set which can be difficult to collect, so most researchers focus on a small number of heavily studied species. The speed with which climate change is progressing necessitates a massive scaling up of the number of species with understood G/P/E dynamics. The research and the knowledge gained in this area will also help human exploration in space, where plants will play an important role (Barker et al., 2023). Previous study has demonstrated that knowledge about one species can be applied to another using ontologically supported knowledgebases that exploit homologous structures and orthologous genes (Naithani et al., 2020). These types of knowledge structures that can apply knowledge about one species to another have the potential to enable the massive scaling up that is needed.

An important part of predicting phenotype is understanding the complex gene regulatory networks present in plants. This study will focus on the promoter region, the 5′ cis-regulatory regions of the homologs. This region is a portion of the DNA strand that is “upstream” from the 5′ end of the gene's coding start site and provides selective binding sites for trans-acting factors such as transcription factors, repressors, and activators that regulate the expression of the gene (Liu et al., 1999). These regions are just one element of the gene expression process. Studying the expression of trans-acting factors is important for understanding the spatiotemporal dynamics of molecular interactions that help adapt or overcome stress. Resources such as the Gene Ontology (The GO Consortium, 2021), Planteome (Cooper et al., 2018), Plant Reactome (Naithani et al., 2020), and KnetMiner (Hassani-Pak et al., 2021) contain much of what we know about gene function, gene regulatory networks, and phenotypes in the form of Gene X regulates Gene Y and Gene Y impacts phenotype Z, but the contextual effect of environmental conditions under which these interactions happen is almost always not included in the annotations. Not all plants and their genes are characterized in detail, but if it is included, the environmental context is usually detailed only in the metadata. Investigations that use protein domain identification and gene homology-based methods to infer the functional role a gene carries out in a given species may be overlooking the spatial and temporal dynamics of mRNA expression that determines whether a gene product (protein) will be present at the desired time and place to serve a molecular function. The interactive nature of genes, environments, and phenotypes requires a data structure that can represent qualitative relationships (e.g., “has phenotype” or “regulates”) and integrate heterogeneous data types in a single, queryable framework. One of these data structures is a knowledge graph (KG) (Sheth et al., 2019).

A graph is made up of objects (nodes) and the relationships (edges) between those objects and, in this context, represents what we know about how biological and environmental entities (objects) interact. Rather than store data in a table or database, a knowledge graph stores the synthesized knowledge we gain from the data, e.g., Gene X has phenotype Y. As more knowledge is added to the graph, more complex queries, network analyses, and inferences can be made. Important examples include the use of knowledge graphs in rare disease diagnosis in humans (Zemojtel et al., 2014), drug repurposing (Reese et al., 2021), improving cancer treatment (Gogleva et al., 2022), and meta-analyses (Tiddi et al., 2020). KGs used for translational science rarely contain environmental exposures even though we know environmental conditions are an important part of gene expression dynamics. The exact way to model exposures in a KG is still under development (Chan et al., 2023). A KG containing information about plant genomics and phenomics under different environmental conditions can be used to generate hypotheses *in silico* for targeting, thereby reducing the number of *in vivo* experiments that need to be conducted, saving time and resources.

This study examines gene expression patterns in response to drought conditions in four plant species, such as *Arabidopsis thaliana, Zea mays, Sorghum bicolor*, and *Populus trichocarpa*. The central motivation of this study is to assess the feasibility of using homologs to make predictions about gene expression in multiple species.

## Materials and methods

### Data description

#### Planteome

The Planteome (https://planteome.org/) is a centralized web portal with a suite of interrelated ontologies for plants and a database of plant genomics data, annotated to the ontology terms (Cooper et al., 2018). In the October 2020 release (version 4.0), the Planteome database included approximately 60,000 ontology terms and more than 3 million data objects, which are connected to ontology terms through approximately 20 million associations. The Planteome database has plant genomic information covering 125 plant taxa. The data available in the Planteome and annotated with ontology terms, include plant gene expression data, traits, phenotypes, genomes, and germplasm sources.

The ontologies developed in-house by the Planteome project include the Plant Ontology (PO; Cooper et al., 2018; Walls et al., 2019), which describes plant anatomical structures and developmental stages, the Plant Trait Ontology (TO) for traits and phenotypes, and the Plant Experimental Conditions Ontology (PECO), which describes experimental conditions and plant exposures. In addition to these, the Planteome hosts the collaborator reference ontologies—the Gene Ontology (GO; The GO Consortium, 2021), Phenotype and Trait Ontology (PATO; Gkoutos et al., 2018), and also a number of species-specific trait dictionaries developed by the Crop Ontology (CO; Shrestha et al., 2010; Arnaud et al., 2020). In the current release, the Planetome includes 11 of the CO trait dictionaries, mapped to the TO.

GO annotations were computationally generated for new species using InParanoid and InterProScan (Shulaev et al., 2011; Myburg et al., 2014). InParanoid was used to predict gene orthology based on the *Arabidopsis thaliana* associations generated by TAIR (Reiser et al., 2022). InterProScan was used to add GO annotations to genes via inference by analyzing protein families and domain mappings (Paysan-Lafosse et al., 2023).

#### EMBL-EBI expression atlas

The EMBL-EBI Expression Atlas (GXA) can be accessed online and is part of the European Bioinformatics Institute (Papatheodorou et al., 2020). It contains manually curated and analyzed data from over 900 plant experiments that have been re-analyzed using the latest versions of the reference plant genome assembly and annotations and by deploying a standardized analysis workflow. Every experiment is fully documented with metadata and provenance.

Gene expression data were downloaded as a table from GXA after searching for desired species and environmental conditions. Data were filtered to include only genes that had statistically different gene expressions (p <0.05) compared with a baseline that was <-1 or >1. Genes with positive differential expression were annotated as having increased expression. Genes with negative differential expression were annotated as having decreased expression. The tabulated data were annotated with additional ontology terms where appropriate and made available in GitHub for graph construction.

### Creating the graph

The graph was created by combining data from Planteome, the GXA, PO, TO, GO, and PECO using the tools available at KG-Hub (Caufield et al., 2023). First, the data and mapping files were downloaded from their respective data repositories. GO-Basic and NCBI Tax-Slim were downloaded from the OBO Foundry in javascript object notation (JSON) format. PO and TO were downloaded from the OBO Foundry in owl format and transformed to JSON using ROBOT (Jackson et al., 2019). Data files containing information about *Sorghum bicolor, Zea mays, Oryza sativa, Populus trichocarpa*, and *Arabidopsis thaliana* were downloaded from Planteome servers in GAF format. Data files containing differential gene expression data involving *Sorghum bicolor, Zea mays, Oryza sativa, Populus trichocarpa*, and *Arabidopsis thaliana* in drought and saline environments were downloaded from the GXA. Several mapping files were used to normalize gene and trait identifiers. Rice gene identifiers were mapped to *Oryza sativa* v7.0 using the ID converter file from the Rice Annotation Project Database (Ouyang et al., 2007; Sakai et al., 2013). Maize gene identifiers were mapped to Zm-B73-REFERENCE-NAM-5.0 assembly using a mapping file that includes all B73 assembly versions and includes the DAGchainer analysis which was obtained from MaizeGDB (Portwood et al., 2019; EMBL-EBI). Poplar gene identifiers were mapped to the reference genome using a mapping file from Gramene (Tello-Ruiz et al., 2018). *Sorghum* gene names were normalized to *Sorghum bicolor* v3.1.1 (McCormick et al., 2018). Plant traits and phenotypes were annotated with TO terms using a look-up dictionary file. Second, each of the data files was transformed into standardized nodes and edges in a tsv file using custom scripts. These scripts normalized gene and trait identifiers using ontologies and the provided mapping files and annotated every entity with a Biolink semantic type (Table 1), and relationships between entities were described using Biolink predicates (Table 2). The graph was assembled according to the Biolink model, which provides standard semantic types and relationships for biological entities (Unni et al., 2022).

**Table 1:** Identifiers and Biolink semantic types assigned to elements of the graph.

**Biological element**	**Identifier**	**Biolink type**
Plant part	PO	Anatomical entity
Growth stage	PO	Life stage
Plant trait	TO	Phenotypic feature
*Zea mays* gene	Zm00001eb IDs	Genomic entity
*Sorghum bicolor* gene	Sobic IDs	Genomic entity
*Oryza sativa* gene	LOC_Os IDs	Genomic entity
*Populus trichocarpa* gene	POPTR IDs	Genomic entity
Experimental condition	PECO	Environmental exposure
QTL	Gramene IDs	Genomic entity
Cultivar	NCBITaxonomy	Organismal entity
Taxon	NCBITaxonomy	Organism taxon
Cellular component	GO	Cellular component
Molecular function	GO	Molecular function
Biological process	GO	Biological process
Germplasm	GRIN and IRIC IDs	Organismal entity

**Table 2:** Edges and their Biolink predicates.

**Subject entity**	**Predicate type**	**Object entity**
Genomic entity	In taxon	Organism taxon
Genomic entity	Active in	Cellular component
Genomic entity	Regulates	Biological process
Genomic entity	Enables	Molecular function
Genomic entity	Expressed in	Anatomical entity
Genomic entity	Expressed in	Life stage
Genomic entity	Has phenotype	Phenotypic feature
Genomic entity	Orthologous to	Genomic entity
Organism taxon	Has phenotype	Phenotypic feature
Organismal entity	In taxon	Organism taxon
Organismal entity	Has phenotype	Phenotypic feature
Environmental exposure	Increases expression of	Genomic entity
Environmental exposure	Decreases expression of	Genomic entity

There was not enough overlapping expression data to include *O. sativa* or saline environments in this analysis, but they were included in the graph.

**Figure 1:**
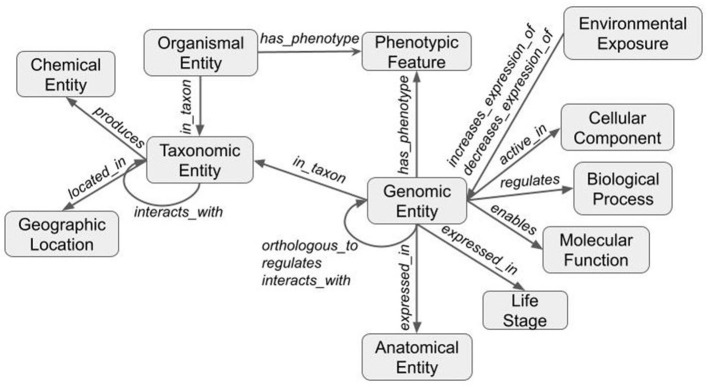
Structure of the knowledge graph. Data are transformed using ontologies and the Biolink model to form a graph. Nodes (gray boxes) are labeled with Biolink semantic type and edges (gray arrows) are labeled with Biolink predicate. Arrows indicate directionality.

**Figure 2:**
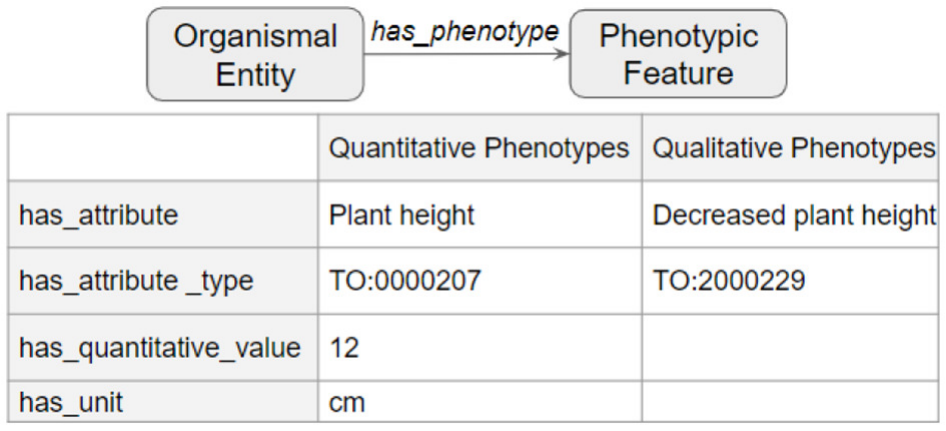
Phenotype data in edge properties. Detailed phenotype information was represented as a collection of edge properties that can accommodate quantitative and qualitative phenotypes.

The third and final step merged the transformed tsv files into a deduplicated list of nodes and edges in KGX format. The final graph consisted of over 400,000 nodes and over 5,000,000 edges and contained additional data from EOLTraitbank that was not used in this study (Figure 1). Specific information about quantitative and qualitative plant phenotypes was represented as an edge property (Figure 2).

### Querying the graph

The merged node file and edge file were uploaded into Neo4j for exploration and query. A Cipher query (Box 1) was used to find all of the homologous genes that had been documented to have differential gene expression in either a drought or a saline environment (Supplementary material 1, 2). The saline environment did not return overlapping data.

Box 1 **Cipher query.**

MATCH (e {id:'PECO:0007404′})-[r]->(g),(g)-[q:‘biolink: orthologous_to‘]-(h), (e {id:'PECO:0007404′})-[s]->(h) RETURN ^*^

Genes returned from the query for the drought environment were compared based on GO annotations (Supplementary material 3), but this also did not give enough data to make conclusions using PANTHER (Supplementary material 4).

### Comparing promoter regions

We collected 5′-regulatory regions of the identified genes (700–900 bp) using BioMart in the Gramene database (Spooner et al., 2012) and searched for potential transcription factor-binding sites using PlantPAN (Chow et al., 2016). Using these data (Supplementary material 5), we created a matrix comparing the occurrence of each transcription factor in the binding site of each gene pair and made note of which were or were not held in common. We used ClustVis (Metsalu and Vilo, 2015) to examine the similarity between the transcription factor-binding sites for each of the *Populus* and *Arabidopsis* gene pairs using PCA. A total of 12 transcription factor-binding sites (AT-Hook, bHLH, C2H2, Dehydrin, Dof, GATA, Homeodomain, Myb/SANT, NF-YB, TBP, Trihelix, and ZF-HD) were present in the promoter regions of all the genes studied and thus were removed from clustering analysis. The same data were fed into Morpheus (Müller et al., 2008) for hierarchical clustering performed with default parameters using One minus Pearson's correlation and complete linkage methods on the TF-binding site annotations. Additional similarity matrices were created using Pearson's correlation metric to separately examine the TF-binding site annotations for genes with similar and contrasting expression profiles. The correlation heatmap colors were adjusted for visualization purposes.

### Data availability

The merged KG data are hosted on the CyVerse DataCommons (https://datacommons.cyverse.org/browse/iplant/home/shared/genophenoenvo). The KG data are available for direct download or remote visualization via CyVerse WebDav service (https://data.cyverse.org/dav-anon/iplant/commons/community_released/genophenoenvo/kg/) using visualization software such as Neo4J. The Python code used to create the graphs is publicly hosted on GitHub (https://github.com/genophenoenvo/knowledge-graph). The final merged KG includes two tab-separated value (tsv) files which include the edges and nodes.

## Results

The graph query returned 62 pairs of homologous genes from *Sorghum bicolor, Zea mays, Arabidopsis thaliana*, and *Populus trichocarpa* (Supplementary material 6), but only 16 pairs between *A. thaliana* and *P. trichocarpa* had documented similar (8) and differential (8) expressions in drought conditions (Table 3). All of the genes with similarly expressed pairs had decreased expression. Expression data for the 16 homologous pairs of *A. thaliana* and *P. trichocarpa* came from two studies in GXA (de Simone et al., 2017; Filichkin et al., 2018).

**Table 3:** Gene expression in *A. thaliana* and *P. trichocarpa* homologous genes under drought conditions.

*A. thaliana* Gene^*^	*P. trichocarpa* Gene^*^	**Gene function (from Planteome)**
AT3G49960 ↓	POPTR_007G053400v3 ↑	Peroxidase activity, response to oxidative stress, heme binding
AT1G70710 ↓	POPTR_010G109200v3 ↓	Catalytic activity, hyrolase activity, carbohydrate metabolic process
AT1G10550 ↓	POPTR_014G115000v3 ↓	Xyloglucan metabolism, hyrolase activity, carbohydrate metabolic process, cell wall biogenesis
AT5G67400 ↓	POPTR_007G053400v3 ↑	Peroxidase activity, response to oxidative stress, heme binding, hydrogen peroxide catabolic process
AT5G23210 ↓	POPTR_005G091700v3 ↓	Proteolysis, serine-type carboxypeptidase activity
AT1G67750 ↓	POPTR_008G182200v3 ↓	Pectate lyase activity, metal ion binding
AT2G39530 ↓	POPTR_010G205300v3 ↑	iron/sulfur cluster binding
AT5G13140 ↓	POPTR_003G167100v3 ↓	Response to nematode, pectate lyase activity, metal ion binding
AT1G11580 ↓	POPTR_011G025400v3 ↑	Enzyme inhibitor activity, pectinesterase activity, cell wall modification, rRNA N-glycosylase activity, aspartyl esterase activity, toxin activity, defense response
AT3G27400 ↓	POPTR_001G339500v3 ↑	Response to nematode, pectate lyase activity, metal ion binding
AT4G02330 ↓	POPTR_014G127000v3 ↑	Enzyme inhibitor activity, pectinesterase activity, cell wall modification, response to stress, aspartyl esterase activity
AT5G20630 ↓	POPTR_006G142600v3 ↓	Manganese ion binding, nutrient reservoir activity
AT4G26260 ↑	POPTR_018G069700v3 ↓	Iron ion binding, inositol oxygenase activity, syncytium formation, L-ascorbic acid biosynthetic pathway
AT2G44990 ↑	POPTR_014G056800v3 ↓	Oxidoreductase activity, secondary shoot formation, carotene catabolic process, strigolactone biosynthetic process, xanthophyll catabolic process, metal ion binding
AT1G70710 ↓	POPTR_010G109200v3 ↓	Cellulase activity, cell wall modification, hydrolase activity
AT1G12940 ↑	POPTR_015G081500v3 ↓	Transmembrane transport

* ↓ Indicates decreased expression and ↑ indicates increased expression.

**Figure 3:**
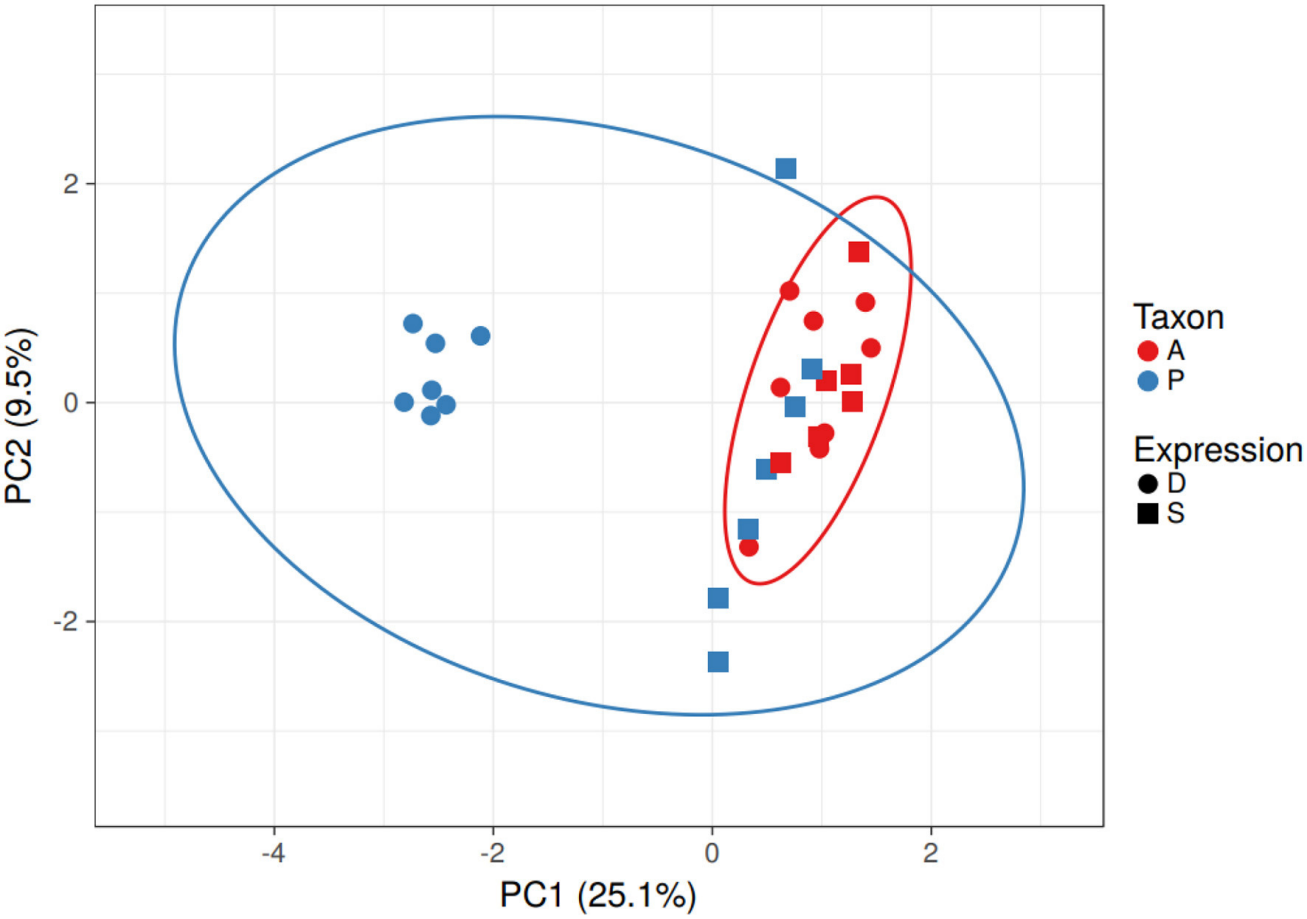
Clustering of *Populus* and *Arabidopsis* genes based on similarity of the transcription factor-binding sites in the promoter region - PCA. The *Populus* genes from the differentially expressed homolog pairs (blue circles) clustered away from the other *Populus* (blue) and *Arabidopsis* (red) genes. Differentially expressed genes are represented as circles and similarly expressed genes are represented as squares. Note that taxonomic differences (blue and red ovals) do not explain the differences in gene expression. No scaling is applied to rows; SVD with imputation is used to calculate principal components. X and Y axes show principal component 1 and principal component 2 that explain 25.1 and 9.5% of the total variance, respectively. *N* = 29 data points.

Based on the predicted transcription factor-binding sites in the promoter regions, the *Populus* genes in the differentially expressed homolog pairs cluster separately from the other *Populus* and *Arabidopsis* genes (Figure 3). This difference is driven by a group of 11 transcription factor-binding sites that are absent in the promoter regions of the subset of divergent *Populus* genes (RAV, MIKC, NAM, G2-like, CPP, ARR-B, tify, TALE, NF-YC, ERF, and NF-YA). The separation of these genes cannot be explained by the taxon or the study providing the data (which overlaps the taxon).

There were seven *Populus* genes that clustered away from the others. All but one (POPTR_014G056800v3 involved in strigolactone biosynthesis) were hypothetical proteins (According to Gramene). GO annotations for these genes clustered around transporter activity, catabolic activity, response to stress, binding, and catalytic activity. The 11 transcription factors absent in the binding sites of the *Populus* genes include proteins involved in plant stress response in *Arabidopsis* (According to UniProt).

**Figure 4:**
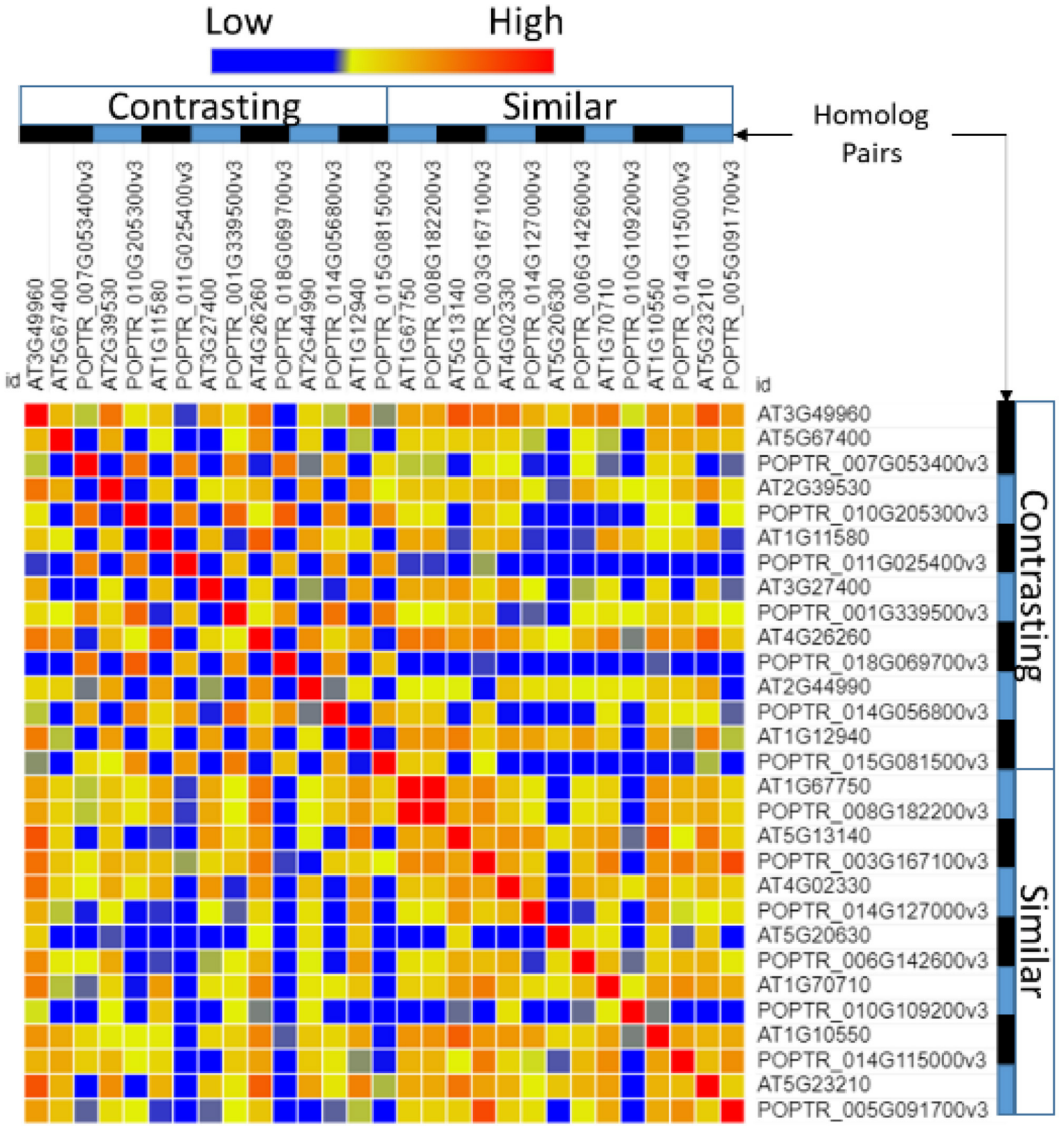
Similarity of the transcription factor-binding sites in the promoter region of *Populus* and *Arabidopsis* homologous gene pairs. Poplar (POPTR) and *Arabidopsis* (AT) genes were grouped into their homolog pairs and whether they had similar or contrasting gene expression when exposed to drought. This figure shows that the promoter regions of pairs with contrasting expressions were less similar (blue) and the promoter regions of pairs with similar expressions were more similar (red).

A comparison of the promoter regions between homolog pairs showed that homologs that were expressed similarly had more similar promoter regions than pairs that were expressed differentially (Figure 4).

**Figure 5:**
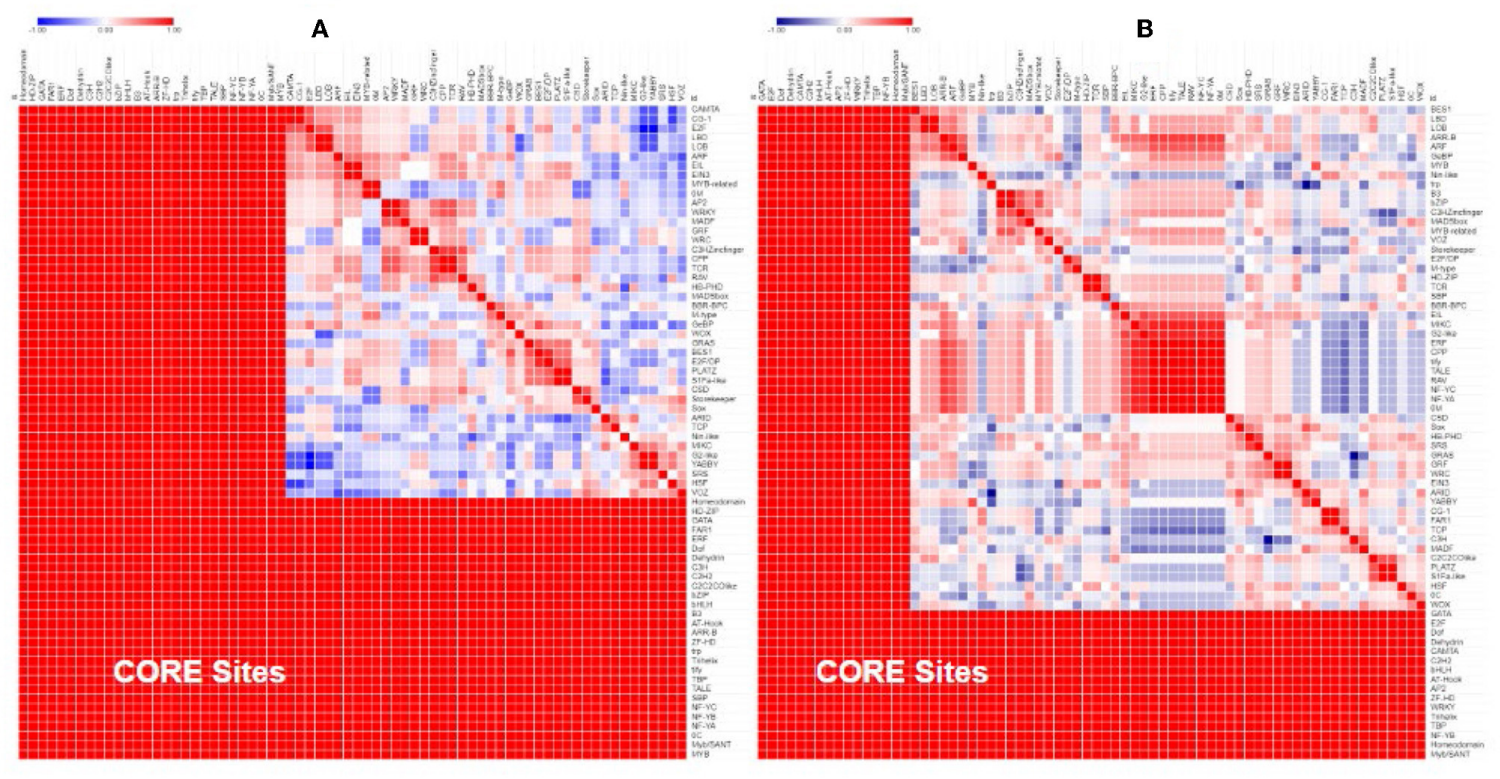
Similarity of the transcription factor-binding sites in the promoter region of *Populus* and *Arabidopsis* genes grouped by their expression profile. Genes that were similarly expressed in a drought treatment **(A)** had more similar promoter regions (red) than genes that were differentially expressed **(B)**.

Separate comparisons of the promoter regions from gene pairs with contrasting expression profiles also show that gene pairs with similar expression had more similar promoter regions (Figure 5A) and gene pairs with contrasting expression had less similar promoter regions (Figure 5B).

## Discussion

This study shows that one can use *in silico* experiments to predict gene expression in drought conditions using homologous gene families in some species pairs but not all. This study supports previous findings that in some cases, promoter regions evolve separately from the coding region of the genes they regulate (Tirosh et al., 2008). Thus, we can translate knowledge about gene expression in one species to another, but we need to include these dynamics in the data infrastructures we use to make this translation, in this case, KGs. Many data structures link a gene to a phenotype, trait, or disease without specific expression information. The current representation of differential gene expression links exposure to a chemical or a drug to the increased or decreased expression of a specific gene in the context of toxicology and drug development (Fecho et al., 2022; Unni et al., 2022). Gene regulatory networks are represented as mini-networks of genes that influence other genes (The GO Consortium, 2021), but many of these networks are still unknown in plants. In the short term, *in silico* KG experiments involving gene expression can be improved by including empirically validated gene expression patterns of homologs.

Gene regulatory networks in plants have been developed using a combination of experimental and computational approaches (Kulkarni and Vandepoele, 2020). Methods combining high-throughput DNA sequencing (ChIP-seq) and expression data have successfully revealed the detailed regulatory networks controlling flowering (Chen et al., 2018) but are difficult to scale. Methods such as ATAC-seq and DAP-seq are more scalable but only reveal a partial picture of the regulatory network (O'Malley et al., 2016; Maher et al., 2018). KGs can be used to infer regulatory networks at scale, but the quality is highly dependent on the data used to build the KG. The advantage of applying a KG is the ability to integrate incredibly heterogeneous data in a single graph, thus modeling regulatory networks in their larger biological context. An example of this application is the relatively new field of “network medicine” that uses KGs to examine the progression of disease (Silverman et al., 2020). The main disadvantage of KGs in this application is that large amounts of computable data and domain-specific knowledge models are needed to create a graph of this type. Many disciplines do not have these resources available. While KGs can infer gene regulatory networks, these networks should always be confirmed using established experimental and computational approaches.

These opposing gene expression patterns are not a concern for researchers who are only interested in finding a list of genes that are potentially important in a specific context. It is not until one needs to generate hypotheses about the impact of the environment on the biological function that more complex graph representations become needed. If we are to incorporate the effect of the environment, we need to know more than that Gene X has phenotype Y. We need to know if the environmental effect increases or decreases the expression of the gene and the biological consequences of that change in expression. In some cases, we may only know that an environment is linked to a specific phenotype without knowing the underlying mechanism. This information can still add useful knowledge to the graph. In some cases, the graph itself can be used to generate hypotheses about the interplay between genes, biological processes, molecular functions, cellular components, and an observed phenotype.

Despite having the graph available to quickly explore the data and locate genes of interest, the workflow for comparing the promoter regions required substantial manual intervention. In this instance, we only had 16 gene pairs to explore, but scaling up these types of analyses will require the ability to traverse data annotated with gene identifiers and gene coordinates. Future studies should include extending the graph model to include these data types.

The semantic representation of the effect of environmental exposure on gene expression is more straightforward for the effects of a chemical or a substance, such as phenol or rubber cement. Data can be collected in the laboratory using model organisms, and the results added to the graph for analysis and translational research. Everyday environmental exposures are rarely this simple and frequently involve exposure to many types of substances in different contexts, such as climate or socioeconomic status. Future studies may need to develop ontologies and semantic representations for these more complex exposures.

Our observations support our hypothesis and justify the extension of our KG to include TF-binding site annotations and the actual TF genes, which are either known empirically or are supported by co-expression network analysis. In future, an investigation of conservation vs. non-conservation of cis- and trans-regulatory regions of genes may improve the understanding of interspecies and intraspecies responses to stress and adaptation.

## Data availability statement

The merged KG data are hosted on the CyVerse DataCommons (https://datacommons.cyverse.org/browse/iplant/home/shared/genophenoenvo). The KG data are available for direct download or for remote visualization via CyVerse WebDav service (https://data.cyverse.org/dav-anon/iplant/commons/community_released/genophenoenvo/kg/) using visualization software such as Neo4J. The python code used to create the graphs are publicly hosted on GitHub (https://github.com/genophenoenvo/knowledge-graph). The final merged KG includes two tab-separated value (tsv) files which include the edges and nodes.

## Author contributions

AT developed and framed research question(s), analyzed data, contributed to data analysis, developed software, contributed to writing and revising the paper, and project administration and management. HH contributed to data analysis. TS contributed to data analysis and contributed to writing and revising the paper. JR developed software, validated results or software, developed and framed research question(s), and contributed to writing and revising the paper. LC contributed to data analysis, project administration and management, and contributed to writing and revising the paper. PJ developed and framed research question(s), analyzed data, contributed to data analysis, and contributed to writing and revising the paper. JE contributed to data analysis, developed software, validated results or software, and contributed to writing and revising the paper. All authors contributed to the article and approved the submitted version.

## Appendix

All supplementary files can be accessed in GitHub under a CC-0 license (https://github.com/diatomsRcool/supplementary_material/tree/main/promoter_region).

1. drought_expression.tsv
2. drought_genes.tsv
3. GO_annotations.tsv
4. panther_results folder
5. promoter_region_clustvis_data0.tsv
6. orthologous_genes.tsv

Clustvis analysis is at https://biit.cs.ut.ee/clustvis/?s=IWJNurmUtGWZoMt.
